# Associations between artificial light exposure during pregnancy and the risk of gestational diabetes mellitus: a prospective cohort study

**DOI:** 10.3389/fendo.2026.1854094

**Published:** 2026-06-01

**Authors:** Shufeng Lin, Shiying Wu, Shuanglong Liu, Jiaxiang Huang, Yaping Xie

**Affiliations:** The Second Affiliated Hospital of Fujian Medical University, Quanzhou, China

**Keywords:** artificial light at night, circadian rhythm, dose-response relationship, electronic device screen time, gestational diabetes mellitus, prospective cohort study

## Abstract

**Background:**

Gestational diabetes mellitus (GDM), characterized by glucose intolerance first identified during pregnancy, poses significant risks to both maternal and neonatal health. With its prevalence rising globally, GDM has become a critical public health issue. Emerging evidence suggests that environmental factors, such as light exposure, may influence GDM risk.

**Objective:**

This study aimed to investigate the associations between exposure to electronic device screen time, indoor and outdoor light during pregnancy, and the risk of developing GDM, providing new insights for GDM prevention and management.

**Methods:**

A prospective cohort study was conducted, enrolling 1,030 pregnant women from five hospitals in Fujian, China, between January and July 2025. Baseline data were collected during the first and second trimesters, and participants were followed until 24–28 weeks of gestation for GDM diagnosis. Multivariate logistic regression was used to assess associations, and dose-response relationships were analyzed.

**Results:**

Among the participants, 180 (17.48%) were diagnosed with GDM. The adjusted multivariate model revealed that both longer indoor light exposure and total electronic device use time were significant independent risk factors for GDM. Dose-response analysis indicated a significant increase in risk when daily electronic device use and indoor light exposure exceeded 3 hours, with a significant synergistic interaction between the two. In contrast, the use of outdoor protective measures demonstrated a strong protective effect.

**Conclusion:**

Indoor artificial light exposure and electronic device use are significant independent risk factors for GDM, demonstrating clear dose-response relationships and a synergistic effect. Outdoor protective measures can effectively reduce GDM risk. These findings suggest that reducing prolonged screen time and indoor artificial light exposure, as well as adopting outdoor protective measures, may be associated with lower odds of GDM. Intervention studies are needed to evaluate whether modifying these exposures can reduce GDM incidence. Future research should focus on elucidating the underlying biological mechanisms and validating the effectiveness of interventional strategies.

## 1 Introduction

Gestational diabetes mellitus (GDM) is defined as glucose intolerance with onset or first recognition during pregnancy, typically diagnosed in the second or third trimester (1). The global prevalence of GDM is increasing annually (2), affecting approximately 6% of pregnancies in Europe and 15% in China (3, 4). GDM is associated with various adverse outcomes for both mother and offspring, including an increased risk of preeclampsia, preterm birth, macrosomia, and a heightened likelihood of future cardiovascular disease and type 2 diabetes for both (2, 5, 6). Therefore, identifying potential risk factors for GDM is crucial for mitigating its short- and long-term health consequences.

The etiology of GDM is complex and not fully understood, but it is known to be associated with lifestyle factors such as diet and environmental exposures (7, 8), among which light exposure has been proposed as a potential risk factor (9). Artificial light is a recognized risk factor for metabolic diseases like diabetes and obesity (10). Light disrupts circadian rhythms in humans and other organisms, thereby influencing various physiological processes and behavioral patterns (11, 12). Studies suggest that exposure to outdoor artificial light at night (ALAN), particularly blue light due to its short wavelength and high energy, can suppress melatonin secretion and affect insulin regulation and adrenal corticosteroid secretion (13–15).

Evidence from cross-sectional and prospective cohort studies has confirmed an association between ALAN and an increased risk of type 2 diabetes (16, 17). Furthermore, exposure to light at night (LAN) in the bedroom has been correlated with impaired glucose metabolism markers in young adults (18). However, most existing research focuses on single factors, such as the impact of indoor light or electronic device use on type 2 diabetes, with studies on GDM remaining scarce. Recent studies have suggested that exposure to ALAN in the first trimester might be associated with an increased GDM risk (19), and others have explored the relationship between pre-bedtime light exposure and GDM (20). Nevertheless, research on other types of light exposure, such as from electronic devices and outdoor light, is still limited, and most existing studies are cross-sectional, unable to infer causality. To address this gap, we designed a prospective cohort study aiming to analyze the potential associations between various light exposures and GDM risk, thereby providing a scientific basis for developing targeted prevention strategies to improve maternal and infant health outcomes.

## 2 Methods

### 2.1 Study design

This prospective cohort study recruited 1,030 pregnant women in their first or second trimester from five hospitals in Fujian Province, China, between January and July 2025. Baseline exposure data were collected via questionnaires upon enrollment, covering general information and light-related exposures. All participants were prospectively followed until 24–28 weeks of gestation, when GDM screening was performed using a standard 75g oral glucose tolerance test (OGTT) (21).

### 2.2 Study population

#### 2.2.1 Inclusion and exclusion criteria

Inclusion Criteria: (1) Age ≥18 years; (2) Gestational age between 1–24 weeks at enrollment; (3) Singleton pregnancy; (4) Provided written informed consent.
Exclusion Criteria: (1) Pre-existing type 1 or type 2 diabetes; (2) Presence of mental or cognitive disorders; (3) Severe complications such as cardiovascular or cerebrovascular diseases.

#### 2.2.2 OGTT diagnostic criteria

GDM was diagnosed at 24–28 weeks of gestation based on the American Diabetes Association (ADA) criteria (21), defined as meeting or exceeding any of the following thresholds: fasting plasma glucose ≥5.1 mmol/L, 1-hour post-load glucose ≥10.0 mmol/L, or 2-hour post-load glucose ≥8.5 mmol/L.

### 2.3 Research tools and data collection

#### 2.3.1 Measurement tools

1. General Information Questionnaire: Collected data on demographic and clinical characteristics, including age, education level, occupation, residence, gestational age, parity, dietary habits, history of hypertension, and family history of diabetes. Clinical data like OGTT values and hypertension status were confirmed by physicians.
2. Light Exposure Questionnaire: This questionnaire assessed: ① Electronic device usage time: Daily usage hours for mobile phones, tablets, televisions, and computers, as well as total daily usage time. ② Other light exposure metrics: Daily hours of exposure to indoor fluorescent; average daily outdoor sunlight exposure hours in summer and winter. ③ Protective behaviors: Use of protective measures (e.g., sun-protective clothing, umbrellas) during outdoor activities.

#### 2.3.2 Data collection process

Trained investigators administered electronic questionnaires via the Questionnaire Star platform. Participants were informed about the study’s purpose, significance, and instructions for completion. All questionnaires were distributed, completed, and submitted on-site. After submission, two researchers independently checked data quality and performed double-blind electronic data entry. Invalid questionnaires with logical inconsistencies or patterned responses were excluded.

### 2.4 Ethical approval

This study was conducted in accordance with the principles of the Declaration of Helsinki and was approved by the Ethics Review Committee of the Second Affiliated Hospital of Fujian Medical University (Approval No [2025]: (532)). All participants provided written informed consent before study initiation.

### 2.5 Statistical analysis

Various statistical methods were employed to comprehensively assess the associations between variables and GDM risk. The Shapiro-Wilk test was used to assess the normality of continuous variables. For non-normally distributed continuous variables (e.g., BMI), the Mann-Whitney U test was used for group comparisons, with results presented as median and interquartile range (IQR). For normally distributed continuous variables (e.g., age, height), the t-test was used, with results presented as mean ± standard deviation. Categorical variables (e.g., employment status, residence) were analyzed using the Chi-square test, and Cramer’s V coefficient was calculated to assess the strength of association. For ordinal variables (e.g., levels of protective equipment use, electronic device usage duration), linear trend tests were conducted to identify dose-response relationships. Univariate logistic regression was used for initial risk assessment of individual variables. Subsequently, multivariate logistic regression models were built, adjusting for demographic variables such as age, gestational week, and education level to control for potential confounders. Restricted cubic splines (RCS) were fitted to model dose-response relationship curves, and piecewise regression analysis was used to identify potential exposure thresholds (e.g., risk saturation points for electronic device use). Additionally, logistic regression models with interaction terms were employed to test the joint effects of electronic device use, indoor light, and outdoor sunlight exposure on GDM, controlling for outdoor protective measures, with results visualized using predictive probability plots. The statistical significance level was set at P < 0.05. All analyses were performed using R software (version 4.3.0, R Foundation for Statistical Computing).

## 3 Results

### 3.1 Comparison of baseline characteristics of the study participants

At the beginning of the study, a total of 1,256 pregnant women were recruited. After applying the inclusion and exclusion criteria, 1,030 were deemed eligible and included in the prospective cohort. All 1,030 participants completed the follow-up and were included in the final analysis. The Mann-Whitney U test and t-test results indicated no statistically significant differences in continuous variables—including gestational age, age, height, pre-pregnancy weight, current weight, pre-pregnancy BMI, and current BMI—between the GDM and healthy control groups (P > 0.05). The median and interquartile range (IQR) distributions were highly similar between the two groups; for instance, the median age was 30–31 years, and pre-pregnancy BMI centered around 21.24-21.41 kg/m² (**Table 1**). This indicates good baseline comparability between the groups in terms of fundamental demographic and physiological characteristics, effectively controlling for the potential confounding effects of these factors on GDM risk.

**Table 1:** Comparison of continuous variables between the GDM and non-GDM groups.

Variable	Non-GDM(N=850)	GDM (N=180)	Z/t	P
Gestational age (weeks)	16.29(5.00)	16.39(4.93)	-1.44^a^	0.15
Age (years)	30.70 ± 4.04	30.78 ± 4.05	-0.25^b^	0.80
Height (cm)	159.92 ± 5.15	159.84 ± 4.97	0.19^b^	0.85
Pre-pregnancy weight (kg)	55.00(12.00)	55.00(10.00)	-0.59^a^	0.55
Current weight (kg)	57.00(13.00)	58.00(12.50)	-0.99^a^	0.32
Pre-pregnancy BMI (kg/m²)	21.24(4.73)	21.41(3.81)	-0.58^a^	0.56
Current BMI (kg/m²)	22.20(4.75)	22.54(4.10)	-1.02^a^	0.31
Average sunlight exposure (hours)⁰	1.00(0.50)	1.00(1.00)	-1.09^a^	0.28
Total electronic device use time (hours)	10.00(3.00)	14.00(5.00)	-10.27^a^	<0.001

Data are presented as median (IQR) or mean ± standard deviation.

^a^ Mann-Whitney U test; ^b^ t-test.

⁰Average of summer and winter exposure.

Chi-square tests showed that all included socio-demographic characteristics (e.g., employment status, marital status) and clinical history variables (e.g., hypertension, family history of diabetes) were not significantly associated with GDM occurrence (all P > 0.05). Notably, all Cramer’s V coefficients were below 0.1, further indicating a lack of meaningful association strength between these variables and GDM risk (**Table 2**).

**Table 2:** Association analysis of categorical variables with GDM.

Variable	χ²	P	Cramer’s V
Employment status	1.45	0.23	-0.04
Marital status	1.04	0.60	0.03
Education level	2.99	0.70	0.05
Hypertension	0.14	0.71	0.01
Family history of diabetes	0.01	0.94	0.002
History of cesarean section	0.002	0.97	0.001
Exercise habit during pregnancy	0.99	0.32	0.03
Healthy diet	0.59	0.75	0.02
Residence	0.40	0.53	-0.02
Parity	2.96	0.40	0.05

### 3.2 Association between behavioral exposure factors and GDM risk: univariate analysis

Behavioral exposure factors demonstrated clear dose-response relationships. In the trend tests for ordinal variables, the use of protective measures showed a significant negative dose-effect, indicating a stepwise decrease in GDM risk with increasing levels of protection. In contrast, electronic device exposure showed a positive gradient effect: usage durations of mobile phones, tablets, televisions, and computers were all significantly positively associated with risk (all P < 0.001), with mobile phone exposure having the largest effect size (β = 0.1700), suggesting that frequently used mobile devices may be a key risk factor (**Table 3**).

**Table 3:** Linear trend tests for ordinal variables and GDM.

Variable	β	SE	Z	P
Use of outdoor protective measures	-0.16	0.01	-14.28	<0.001
Summer sunlight exposure time	0.005	0.008	0.60	0.55
Winter sunlight exposure time	0.003	0.01	0.30	0.76
Indoor fluorescent light exposure time	0.15	0.02	7.87	<0.001
Mobile phone use time	0.17	0.02	7.83	<0.001
Tablet use time	0.04	0.01	3.83	<0.001
Television use time	0.07	0.01	4.64	<0.001
Computer use time	0.08	0.03	2.85	0.004

Univariate logistic regression analysis revealed that both indoor light exposure time and various electronic device usage times were significantly associated with GDM risk. Each additional hour of daily indoor light exposure was associated with a 61% increase in GDM risk (OR = 1.61, 95% CI: 1.43-1.83, P < 0.001). Electronic device use also showed significant harmful effects, with mobile phone use carrying the highest risk (OR = 1.51, 95% CI [1.36-1.69]), followed by tablet use (OR = 1.33, 95% CI [1.14-1.55]) and television use (OR = 1.30, 95% CI [1.16-1.45]). Computer use, while still significant, had a relatively lower risk (OR = 1.10, 95% CI [1.03-1.17]).The use of outdoor protective measures demonstrated a significant protective effect. Compared to no protection, using protection only in summer (OR = 0.02, 95% CI [0.01-0.04]) and using protection daily (OR = 0.02, 95% CI [0.01-0.03]) both reduced GDM risk by approximately 98% (P < 0.001), with little difference in effect between the two protective patterns.

Notably, average outdoor sunlight exposure time was not significantly associated with GDM risk (OR = 1.06, 95% CI [0.84-1.33], P = 0.63), suggesting that outdoor sunlight exposure per se may not be an independent risk factor, and its effect might be modulated by exposure thresholds or seasonal factors (**Table 4**).

**Table 4:** Univariate logistic regression analysis of light exposure, electronic device use, and GDM risk.

Variable	OR(95%CI)	P
Average sunlight exposure time	1.06 (0.84–1.33)	0.63
Indoor light exposure time	1.61 (1.43–1.83)	<0.001
Total electronic device use time	1.43 (1.34–1.54)	<0.001
Mobile phone use time	1.51 (1.36–1.69)	<0.001
Computer use time	1.10 (1.03–1.17)	0.01
Tablet use time	1.33 (1.14–1.55)	<0.001
Television use time	1.30 (1.16–1.45)	<0.001
Use of outdoor protective measures		
No protection	1.00(reference)	
Summer only	0.02 (0.01–0.04)	<0.001
Daily	0.02 (0.01–0.03)	<0.001

### 3.3 Independent association between light exposure and GDM: multivariate analysis

After adjusting for potential confounders in the multivariate model, the core findings were further confirmed. Both indoor light exposure time (OR = 1.88, 95% CI [1.56-2.28], P < 0.001) and total electronic device use time (OR = 1.43, 95% CI [1.29-1.59], P < 0.001) remained significant independent risk factors. The use of outdoor protective measures showed a clear dose-response protective relationship: compared to no protection, occasional use (OR = 0.01, 95% CI [0.002-0.01]), use only in summer (OR = 0.01, 95% CI [0.01-0.03]), and daily use (OR = 0.01, 95% CI [0.003-0.02]) all significantly reduced the risk of GDM (all P < 0.001).

Among the control variables, pre-pregnancy BMI (OR = 0.73, 95% CI [0.59-0.92], P = 0.01) and current BMI (OR = 1.36, 95% CI [1.09-1.70], P = 0.01) were statistically significant. Residence (OR = 0.53, 95% CI [0.31-0.91], P = 0.02 for urban vs. rural) and hypertension (OR = 28.7, 95% CI [1.27-649.70], P = 0.04) were also associated with GDM risk.

It is noteworthy that age, gestational week, education level, employment status, marital status, family history of diabetes, history of cesarean section, exercise habits, and dietary habits showed no statistical significance after adjustment (all P > 0.05) (**Table 5**).

**Table 5:** Multivariate logistic regression analysis of factors associated with GDM.

Variable		OR(95% CI)	P
Primary Variables	Average sunlight exposure time	1.21 (0.85–1.71)	0.29
	Indoor light exposure time	1.88 (1.56–2.28)	<0.001
	Total electronic device use time	1.43 (1.29–1.59)	<0.001
	Use of outdoor protective measures		
	No protection	1.00(reference)	
	Occasional	0.01 (0.002–0.01)	<0.001
	Summer only	0.01 (0.01–0.03)	<0.001
	Daily	0.01 (0.003–0.02)	<0.001
Control Variables	Age	0.97 (0.91–1.04)	0.45
	Gestational age	0.97 (0.90–1.04)	0.38
	Pre-pregnancy BMI	0.73 (0.59–0.92)	0.01
	Current BMI	1.36 (1.09–1.70)	0.01
	Residence		
	Rural	1.00(reference)	
	Urban	0.53 (0.31–0.91)	0.02
	Hypertension		
	No	1.00(reference)	
	Yes	28.7 (1.27–649.70)	0.04
	Parity		
	1	1.00(reference)	
	2	1.02 (0.50–2.07)	0.96
	3	4.04 (1.35–12.11)	0.01
	4	4.08 (0.51–32.70)	0.19
	Education Level		
	Primary school or below	1.00(reference)	
	Junior high school	0.75 (0.04-13.10)	0.84
	High school or vocational school	1.16 (0.07-20.37)	0.92
	Associate degree	0.94 (0.05-16.42)	0.97
	Bachelor’s degree	0.95 (0.05-16.45)	0.97
	Master’s degree or above	1.15 (0.05-23.95)	0.93
	Employment Status		
	Unemployed	1.00(reference)	
	Employed	1.38 (0.72-2.65)	0.33
	Marital Status		
	Unmarried	1.00(reference)	
	Married	1.48 (0.41-5.41)	0.55
	Family History of Diabetes		
	No	1.00(reference)	
	Yes	0.94 (0.39-2.27)	0.89
	History of Cesarean Section		
	No	1.00(reference)	
	Yes	0.82 (0.35-1.94)	0.66
	Exercise Habit		
	No	1.00(reference)	
	Yes	1.10 (0.65-1.85)	0.72
	Dietary Habit		
	Healthy	1.00(reference)	
	Average	1.14 (0.69-1.88)	0.62
	Unhealthy	0.38 (0.02-6.73)	0.51

### 3.4 Dose-response relationships and exposure thresholds

**Figure 1:**
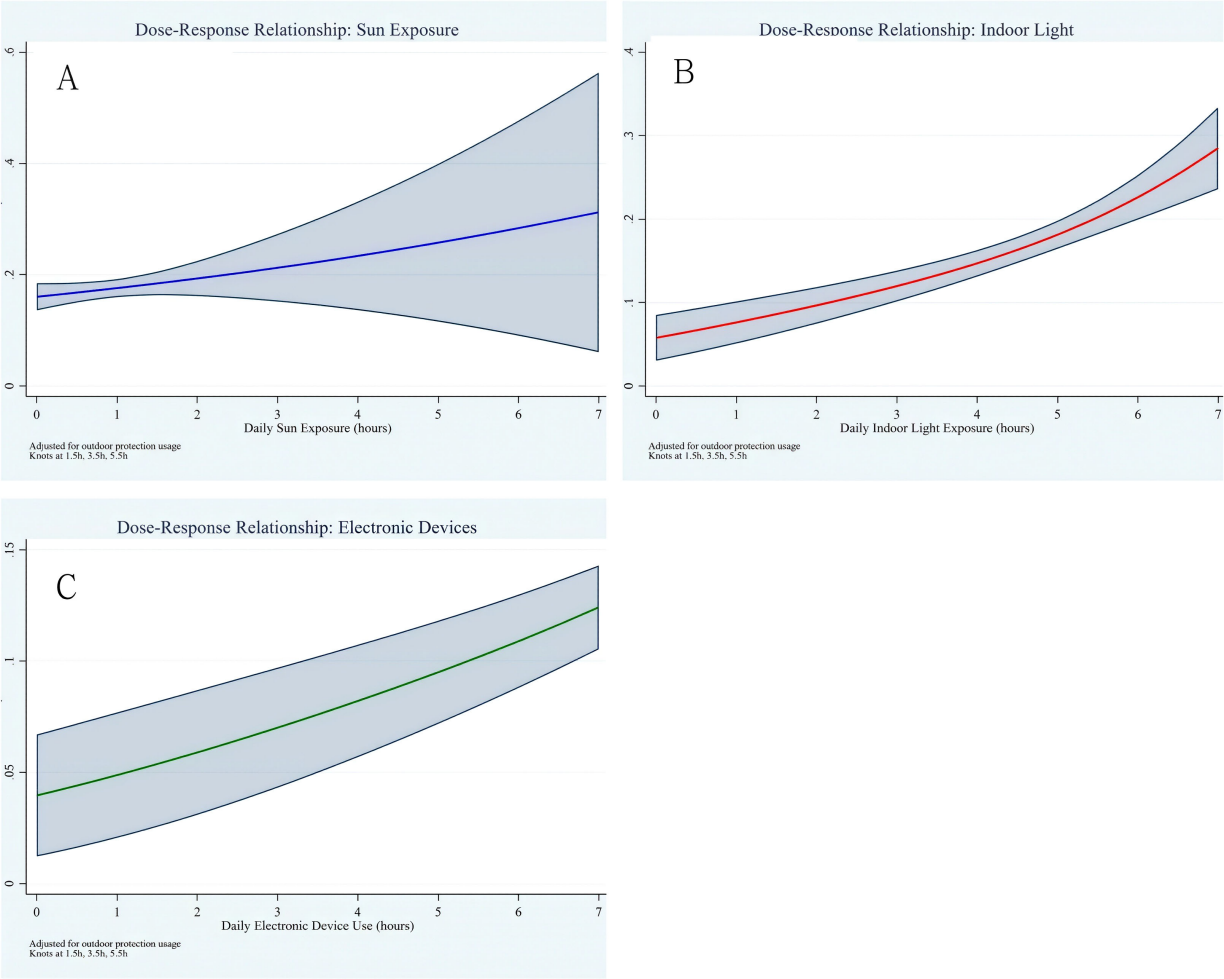
Dose-response curves for different exposure types and GDM risk, fitted using restricted cubic splines. **(A)** Sunlight exposure time, **(B)** Indoor light exposure time, **(C)** Total electronic device use time.

As shown in **Figure 1**, restricted cubic splines visually depicted the dose-response curve characteristics of GDM risk for different exposure types: electronic device use time, indoor light exposure time, and sunlight exposure time. Piecewise regression results are shown in **Table 6**. The risk curve for electronic device exposure showed a clear non-linear characteristic, with an inflection point at 3 hours. For electronic device use exceeding 3 hours per day, each additional hour was associated with a 41% increase in GDM risk (OR = 1.41). The risk curve for indoor light exposure showed a continuously increasing trend. For indoor light exposure exceeding 3 hours per day, each additional hour was associated with a 125% increase in risk (OR = 2.25), with a steeper slope in the latter segment, indicating a more pronounced harmful effect of prolonged light exposure. The risk curve for sunlight exposure showed a J-shaped characteristic: relatively flat in the initial stage, followed by an upward trend in the later stage. However, piecewise regression results indicated no statistical significance for either the ≤3 hours segment (OR = 1.09, P = 0.667) or the >3 hours incremental segment (OR = 1.03, P = 0.968). It should be noted that the 3-hour threshold was derived from exploratory restricted cubic spline and piecewise regression analyses in this single cohort and lacks prior biological or empirical justification. It is therefore a data-driven observation that requires external validation in independent studies before it can be considered a clinically meaningful cut-off. The fitted curves represent exploratory data-driven trends. The confidence intervals widen notably at the extremes of exposure, which indicates some uncertainty in the precise shape of these associations in those regions.

**Table 6:** Piecewise regression results for dose-response relationships.

Variable	OR(95% CI)	P
Total Electronic Device Use Time		
≤3 hours segment	–	–
Increment for >3 hours segment	1.41 (1.29 - 1.54)	<0.001
Indoor Light Exposure Time		
≤3 hours segment	0.83 (0.50 - 1.39)	0.482
Increment for >3 hours segment	2.25 (1.80 - 2.81)	<0.001
Sunlight Exposure Time		
≤3 hours segment	1.09 (0.74 - 1.59)	0.667
Increment for >3 hours segment	1.03 (0.27 - 3.96)	0.968

All models were adjusted for the use of outdoor protective measures.​.

### 3.5 Synergistic effects of combined exposures

**Figure 2:**
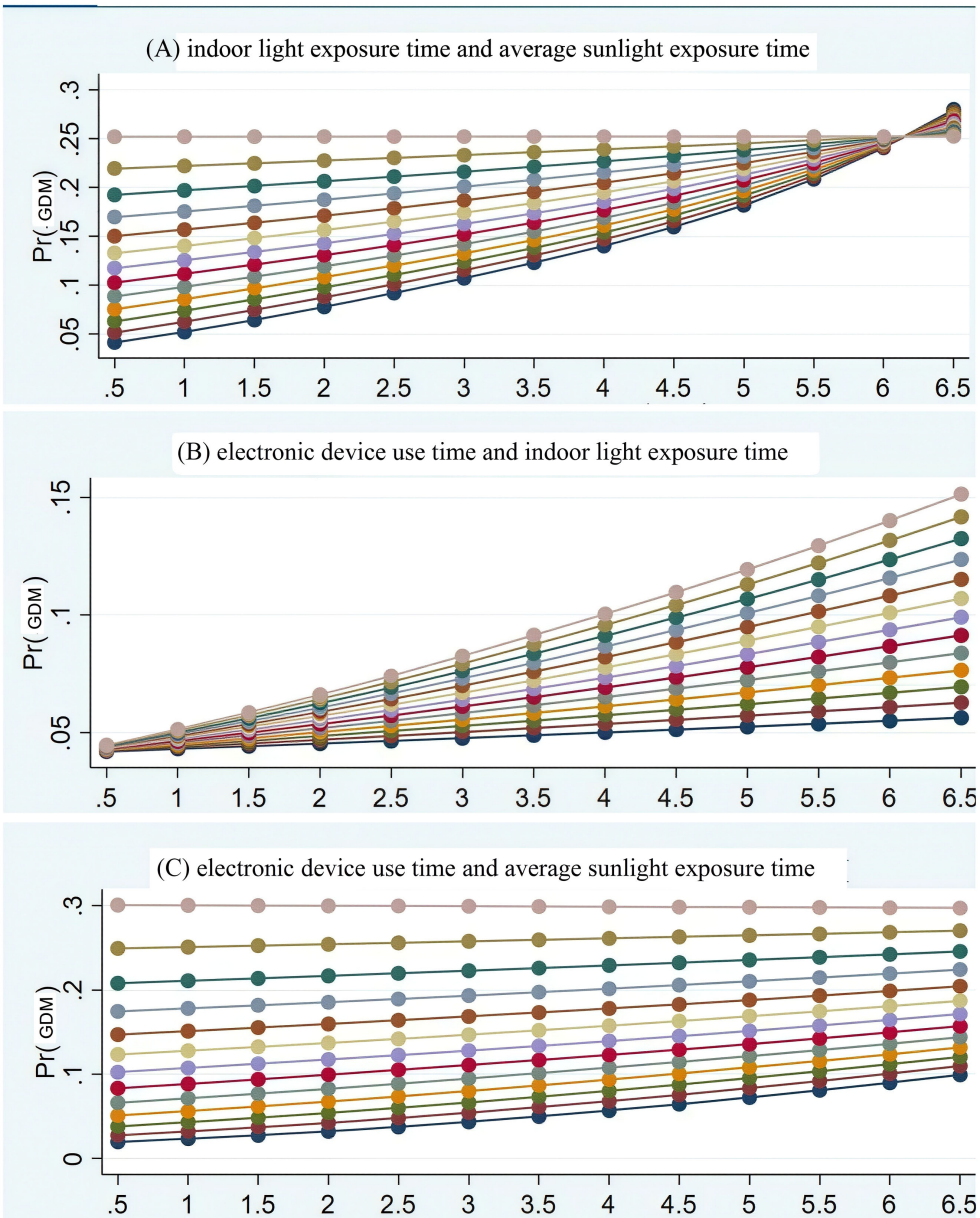
Predictive probability plots for the synergistic effect analysis of combined exposures on GDM risk. The plots visualize the joint effects of **(A)** indoor light exposure time and average sunlight exposure time, **(B)** electronic device use time and indoor light exposure time and **(C)** electronic device use time and average sunlight exposure time.

This study analyzed the interaction effects of electronic device use time, indoor light exposure time, and average sunlight exposure time on GDM risk using logistic regression models (see **Figure 2**, Predictive Probability Plots). The results showed that the interaction between electronic device use time and indoor light exposure time had a significant impact on GDM risk (OR for interaction = 1.055, 95% CI [1.004-1.109], P = 0.034), indicating that as both increase simultaneously, the risk of GDM increases by approximately 5.5%. However, the interaction effects between electronic device use time and average sunlight exposure time (OR for interaction = 0.938, 95% CI [0.821-1.072], P = 0.349) and between indoor light exposure time and average sunlight exposure time (OR for interaction = 0.897, 95% CI [0.708-1.136], P = 0.368) were not statistically significant. These findings suggest that when assessing GDM risk, the superimposed effects of electronic device use time and indoor light exposure time warrant particular attention, highlighting the importance of simultaneously controlling both screen-based and artificial lighting exposures in indoor environments. This study provides a visual scale for clinical intervention. The predictive probability plots should be interpreted as descriptive visualizations of model-based interaction effects, with uncertainty in the underlying estimates reflected in the confidence bounds.

## 4 Discussion

This study is among the first to systematically investigate the associations between various types of light exposure during pregnancy and GDM risk within a prospective cohort design. The main results indicate that both indoor artificial light exposure time and electronic device use time were significantly associated with GDM risk, exhibiting clear dose-response relationships and a significant synergistic effect when co-exposed. These findings offer a new perspective on the impact of light exposure on GDM and provide a scientific basis for GDM prevention and management.

### 4.1 Principal findings

This study found a significant positive correlation between indoor artificial light exposure time and GDM risk. After multivariate adjustment, each additional hour of daily indoor light exposure was associated with an 88% increase in GDM risk (OR = 1.88). The strength of this association even exceeded that of electronic device use. Dose-response analysis revealed a sharp increase in risk after exposure exceeded 3 hours/day (piecewise OR = 2.25). Importantly, this threshold should be viewed as exploratory and hypothesis-generating, not as a validated guideline for behavioral recommendations. Secondly, electronic device use time was significantly associated with GDM risk, with mobile phone use showing the strongest association (unadjusted OR = 1.51). Dose-response analysis indicated that for electronic device use exceeding 3 hours per day, each additional hour was associated with a 41% increase in GDM risk (piecewise OR = 1.41).

In this cohort, outdoor protective measures were associated with a substantially lower odds of GDM (OR = 0.02). However, given the observational design and the unusually large point estimate, this association may partly reflect unmeasured behavioral or socioeconomic confounding (e.g., women who regularly use sun protection may also practice other health-conscious behaviors) rather than a direct biological effect of protection alone. The magnitude of this association should be interpreted with caution and requires replication in independent populations. This suggests that the risk may not stem from outdoor activity itself, but rather from unprotected exposure to ultraviolet and blue light. This result is consistent with the finding that outdoor sunlight exposure time per se was not significantly associated with GDM, highlighting the critical role of the “mode of exposure” rather than the “exposure behavior” itself. Future research should employ interventional trials to investigate whether appropriate protective measures (e.g., using sunscreen, umbrellas) can mitigate the potential disruption of abnormal light signals to the circadian system while preserving the benefits of outdoor activity (e.g., promoting vitamin D synthesis, alleviating psychological stress).

Notably, our analysis of synergistic effects from combined exposures revealed a significant interaction between electronic device use time and indoor light exposure time on GDM risk (OR for interaction = 1.055, P = 0.034). While the interaction between electronic device use and indoor light exposure was statistically significant, its magnitude is modest, and its clinical relevance at the individual level remains uncertain. Rather, this finding raises the possibility of a biological interaction that deserves further mechanistic investigation in future studies. From a public health perspective, this reveals a compound risk associated with modern lifestyles: many pregnant women may experience high illumination in indoor environments during the day and continue using electronic devices in the evening. This “dual exposure” pattern may exert additive adverse effects on glucose metabolism. This finding underscores the importance of concurrently controlling both screen-based and artificial lighting exposures in indoor environments. While the direction and dose-response pattern for indoor light exposure is robust, the precise magnitude of the odds ratio (OR = 1.88 per additional hour) should be confirmed in studies with objective exposure measurement before being considered a definitive risk estimate.

An unexpected finding was the lack of statistically significant associations for several well-established GDM risk factors in our multivariate model (e.g., family history of diabetes, advanced maternal age). This may reflect limited statistical power, the specific characteristics of our study sample, or the way certain variables were categorized. It should not be interpreted as evidence against the established roles of these risk factors, and future studies with larger or more diverse samples are needed to re-examine these relationships in the context of light exposure.

### 4.2 Comparison with previous studies and potential mechanisms of novel findings

Our findings are generally consistent with previous studies that reported an association between exposure to nighttime artificial light (LAN) and an increased risk of type 2 diabetes (16, 17, 22), but this study extends the evidence to the field of GDM and is the first to detail and report the independent and joint effects of different types of light exposure (indoor fluorescent light, electronic devices, outdoor sunlight) and protective behaviors. Previous studies on GDM and light have mostly focused on mid-to-late pregnancy (19) or pre-sleep light exposure (20), with relatively limited evidence. Our study, covering exposure in early and mid-pregnancy, suggests that the risk window for light exposure on GDM might begin earlier. We did not find a significant association between sunlight exposure and GDM, which aligns with some studies suggesting moderate sunlight exposure benefits metabolic health (23), but also implies that such benefits may depend on effective protection.

Both indoor lighting and electronic device use in our study were significantly associated with GDM risk. Indoor artificial light sources (especially blue light-rich LED lighting) and electronic device screens are major sources of evening blue light exposure (24). Studies indicate that artificial blue light exposure can significantly suppress melatonin secretion (by up to 50%) and delay circadian phase (25). Melatonin plays a crucial role in blood glucose regulation. Beyond regulating sleep-wake cycles and plasma leptin rhythms (12), melatonin receptors are widely distributed in pancreatic β-cells and hepatocytes, directly involved in insulin secretion and glucose homeostasis regulation (26, 27).

An experimental study on healthy adults found that blue light-enriched light exposure increased insulin resistance and blood glucose peaks, providing direct human evidence for its potential detrimental effect on glucose metabolic function (28). This mechanistic pathway is further supported by animal studies. Research in animals indicates that nighttime light exposure impairs glucose tolerance and significantly decreases plasma insulin in a time-, intensity-, and wavelength-dependent manner (29, 30). Some studies suggest that light can acutely impair glucose tolerance in mice by activating intrinsically photosensitive retinal ganglion cells (ipRGCs) that project to the suprachiasmatic nucleus (SCN). This effect implicates a neural circuit from the SCN to the brown adipose tissue (BAT), wherein light activation blocks BAT adaptive thermogenesis, preliminarily revealing a retina-SCN-BAT axis. This preliminarily reveals the retina-SCN-BAT axis as a potential rapid pathway for light-mediated glucose metabolism regulation, although its role in human pregnancy requires further elucidation (10).

Further animal studies also provide further biological plausibility for these associations. In a rat model, disrupting maternal circadian rhythms through chronic phase shifts of the photoperiod during gestation has been shown to severely alter the circadian profiles of plasma glucose, insulin, and other metabolic hormones, leading to poor fetal metabolic programming (31). Similarly, a mouse model demonstrated that environmental circadian disruption across the lifespan impairs glucose tolerance and induces insulin resistance in adult mice, effects that were associated with impaired insulin signaling/AKT in skeletal muscle and liver (32). Additional mechanistic evidence comes from a study using female golden hamsters, which revealed that a light shift (rotating light schedule) activated the PKC-vated pathway and simultaneously altered the rhythmic expression of core circadian genes such as Aanat and Bmal1 (33). A separate investigation in the same animal model further showed that long-duration light exposure during pregnancy leads to circadian desynchronization of the central and peripheral reproductive clocks, an effect mediated through the Akt/FoxO1 signaling pathway and associated with adverse uterine physiology and pregnancy outcomes (34). Collectively, these animal findings demonstrate that chronic light-induced circadian disruption can dysregulate key molecular pathways—including Bmal1, Aanat, Akt, and FoxO1,ysrt are intricately linked to both circadian rhythms and metabolic homeostasis. Although these mechanisms were not directly assessed in our study, they offer a plausible biological framework for understanding how prolonged artificial light and electronic device use during pregnancy could adversely affect maternal glucose metabolism. Because melatonin levels, circadian phase markers, and other relevant biomarkers were not directly measured in this study, the proposed pathways remain speculative. Future studies incorporating these biomarkers are essential to test whether light-induced circadian and melatonin disruption indeed mediates the observed associations with GDM.

Beyond the biological mechanisms discussed above, the magnitude of the protective association for outdoor measures also warrants scrutiny. The unexpectedly strong protective associations observed for outdoor protective measures should be interpreted with caution, as they contrast with the more modest protective associations typically reported in lifestyle-metabolism research. These estimates may partly reflect unmeasured behavioral or socioeconomic confounding (e.g., women who habitually use sun protection may also differ in diet, physical activity, or healthcare-seeking behaviors). Such confounding could exaggerate the apparent protective association, and independent replication in cohorts with more comprehensive behavioral phenotyping is needed to verify these findings.

### 4.3 Strengths and limitations

This study has several limitations. First, data on light exposure and electronic device use were collected exclusively through self-reported questionnaires, which are susceptible to recall and reporting bias and may lead to non-differential or differential misclassification of exposure. Because no objective validation was performed (e.g., personal light sensors, device screen-time logs), the magnitude of the observed associations may be affected. Future studies could employ objective monitoring devices, such as light sensors and screen time tracking software, to enhance exposure assessment accuracy. Second, although numerous known confounders were adjusted for, residual confounding from unmeasured variables—particularly sleep duration and quality, detailed dietary composition, and intensity of physical activity, all of which are central to circadian and metabolic regulation—cannot be ruled out and may have influenced the observed associations. Future prospective studies should incorporate validated instruments for these domains. Furthermore, participants were recruited from only five hospitals, potentially limiting the generalizability of the findings, which need validation in other regions and populations.

Despite these limitations, the prospective cohort design provides valuable evidence for the temporal associations between exposure and outcome compared to the cross-sectional designs prevalent in previous research. Furthermore, various supplementary analyses (e.g., dose-response, interaction analysis) enhanced the robustness of the findings. Nevertheless, given these limitations, the current findings are best viewed as hypothesis-generating. Clinical recommendations cannot be derived from this single observational study, and well-designed intervention trials are required to translate these associations into practice.

### 4.4 Potential areas for future investigation and public health considerations

From a clinical perspective, the findings may also be relevant to perinatal outcomes beyond maternal glucose control. GDM is an established risk factor for adverse neonatal outcomes, including increased susceptibility to infections. These complications frequently necessitate additional medical interventions such as prolonged hospitalization and intensified monitoring; early antibiotic exposure, a practice commonly documented in neonatal care settings (35), may also occur. Therefore, if the observed associations are confirmed in future intervention studies, optimizing light exposure behaviors during pregnancy might not only influence maternal glucose metabolism but also potentially reduce downstream neonatal complications.

Behavioral Interventions for Light Exposure: Based on the current findings, the following areas warrant further investigation before any clinical recommendations can be made: Future intervention studies could evaluate whether optimizing indoor lighting (e.g., reducing evening blue-enriched light) and limiting recreational screen time during pregnancy reduces GDM incidence.
Health Education: If confirmed, knowledge about “light health” could be integrated into prenatal education programs.
Future Research Directions: Subsequent studies should focus on: a) elucidating the biological mechanisms linking light exposure with GDM, for instance, by assessing the rhythms of physiological markers such as melatonin and cortisol; b) developing and applying more objective methods for assessing light exposure; and c) ultimately, designing and conducting intervention trials to verify whether behavioral modifications aimed at optimizing light exposure can effectively reduce the incidence of GDM.

## 5 Conclusion

In summary, this prospective cohort study found that indoor artificial light exposure and electronic device use during pregnancy were independently associated with higher odds of GDM, with evidence of a synergistic interaction. The use of outdoor protective measures was associated with lower odds of GDM. Given the observational design and limitations in exposure measurement, these findings should be treated as hypothesis-generating rather than confirmatory evidence of causation.

## Data Availability

The raw data supporting the conclusions of this article will be made available by the authors, without undue reservation.
